# P-1342. Morganella morganii: Genomic Insights into Antimicrobial Resistance and Clonal Transmission

**DOI:** 10.1093/ofid/ofaf695.1530

**Published:** 2026-01-11

**Authors:** Nure Sharaf Nower Samia, Md Mobarok Hossain, Fahmida Chowdhury, Shovan Basak Moon, Arefeen Haider, Shahriar Islam, Syeda Mah-E-Muneer, Mohammad Jubair, Mst Noorjahan Begum, Mustafizur Rahman

**Affiliations:** International Centre for Diarrheal Disease Research, Bangladesh, Dhaka, Dhaka, Bangladesh; International Centre for Diarrheal Disease Research, Bangladesh, Dhaka, Dhaka, Bangladesh; icddr,b, Dhaka, Dhaka, Bangladesh; International Centre for Diarrheal Disease Research, Bangladesh, Dhaka, Dhaka, Bangladesh; International Centre for Diarrheal Disease Research, Bangladesh, Dhaka, Dhaka, Bangladesh; International Centre for Diarrheal Disease Research, Bangladesh, Dhaka, Dhaka, Bangladesh; icddr,b, Dhaka, Dhaka, Bangladesh; International Centre for Diarrheal Disease Research, Bangladesh, Dhaka, Dhaka, Bangladesh; International Centre for Diarrheal Disease Research, Bangladesh, Dhaka, Dhaka, Bangladesh; International Centre for Diarrheal Disease Research, Bangladesh, Dhaka, Dhaka, Bangladesh

## Abstract

**Background:**

*Morganella morganii* is an opportunistic pathogen increasingly associated with antimicrobial resistance (AMR), posing a growing clinical threat.

**Figure:**
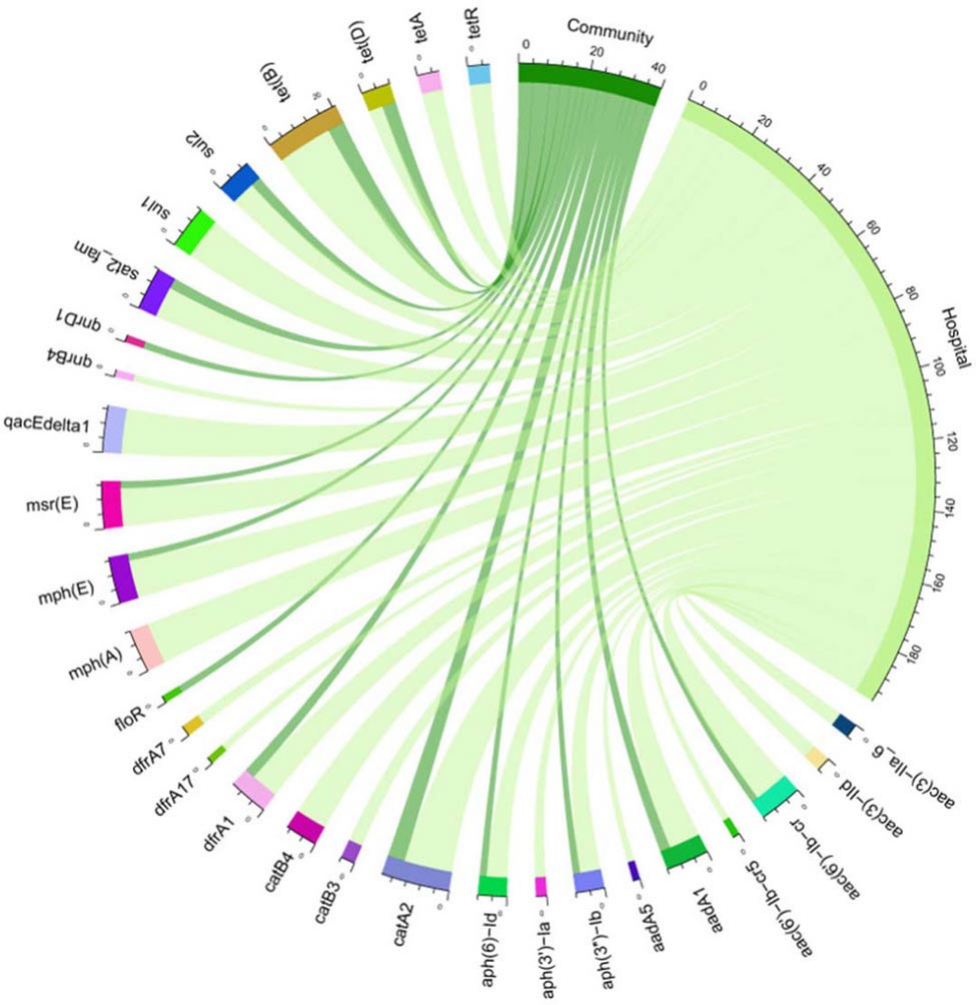
Distribution of Antimicrobial Resistance (AMR) Genes in Community and Hospital Isolates The circular diagram illustrates the distribution of AMR genes across community and hospital isolates, highlighting the presence of tet, sul2, catA2, and aadA1 in both settings. Hospital isolates exhibit the highest diversity with 195 AMR genes, while no known CrE or ColRE determinants were identified in either group.

**Methods:**

We analyzed 47 *M. morganii* strains isolated from hospital patients and healthy community members in urban Dhaka through the Antibiotic Resistance in Hospital and Community (ARCH) study (April–October 2019). Of 2519 stool samples, 47 isolates were confirmed via whole-genome sequencing, despite initial Vitek misidentification of three (8%) isolates as *Escherichia coli*.

**Results:**

High phenotypic resistance was observed: 87% (n=41) carbapenem-resistant (CRE), 8.5% (n=4) extended-spectrum cephalosporin-resistant (EsCRE), and 4.2% (n=2) colistin-resistant (ColRE), although no known genetic determinants explained CRE or ColRE. All isolates were cephalosporin-resistant. Additional resistance included aminoglycosides—amikacin, kanamycin, and tobramycin (29.4%, n=14)—macrolides (31.9%, n=15), tetracycline (46.8%, n=22), streptomycin (42.3%, n=20), and trimethoprim (44.6%, n=21). Detected resistance genes included *mph(A), mph(E), msr(E), erm(B), aph(3'')-Ib, aadA,* and *aph(6)-Id*. Plasmid markers were present in 31.9% (n=15) of isolates (53.3% Inc types, 46.6% Col types), with hospital-derived strains more frequently carrying *tet, sul2, catA2,* and *aadA1* genes. Phylogenetic analysis revealed clonal transmission of CRE, EsCRE, and ColRE strains between hospital and community settings. Pangenome analysis demonstrated significant genomic diversity among isolates.

**Conclusion:**

*Morganella morganii*, once a rare opportunist, accounted for most of the 10% other isolates, highlighting its emerging role in antimicrobial resistance alongside the dominant 90% of *E. coli* and *Klebsiella*. Therefore, enhanced surveillance of MDRO *Morganella morganii* in ICUs is critical. Antimicrobial susceptibility testing should guide treatment, avoiding beta-lactams due to widespread resistance. Aminoglycosides or fluoroquinolones, with combination therapy for severe cases, are options for susceptible strains. This study underscores *M. morganii*’s role in AMR spread and the need for accurate identification and targeted therapies.

**Disclosures:**

All Authors: No reported disclosures

